# Hemorrhagic, or Malignant Bilious Fever

**Published:** 1874-06

**Authors:** W. F. DeWitt

**Affiliations:** Atlanta, Ga.


### HEMORRHAGIC, OR MALIGNANT BILIOUS FEVER.

By W. F. De Witt, M.D.

*Read before the Atlanta Academy of Medicine, May* 4, 1874.

On the 15th day of September, 1869, was called to see Mrs. B., who was suffering with high fever; she had been having chills. This fever came on during the night, after a severe chill. Supposed I had a case of simple bilious fever to treat, and adopted the usual plan. She suffered with constant nausea and vomiting. Matters vomited were blue, like indigo water. Watched for a remission, which was not marked; gave quinine freely; fever recurred as violently as at first; the remissions became less marked, and irregular. She would complain of flushes of heat, and again of cold sensations. There was evident torpor of the liver. Applied a large blister, extending from the left of the umbilicus well around the right side, over the region of the liver. It drew well; serum nearly black. No benefit seemed to be derived from the blister. She took light nourishment regularly. Began to assume a typhoid type; was listless; tongue dry; mind clear; had not passed urine during the last twenty hours; was not suffering any inconvenience, but I thought I would introduce a catheter, and on withdrawing the vessel used to receive the urine, was amazed at finding more than a quart of liquid, having the appearance of slightly diluted claret wine. I had not heard of the new disease at the time. Her skin was then yellow. On the following day introduced the catheter, and withdrew not quite so much of the same character of liquid. I had no reason to doubt that it was bloody urine. She grew weak rapidly; surface yellow, with dark colored spots or splotches appearing under the skin in various parts of the body. Died three or four days after their appearance. Case was under treatment about ten days. The treatment after first day was with quinine and camphor. After tongue became dry, gave oil turpentine or chlorate potass, beef tea, whisky toddy, etc.; lime water and milk, creasote, etc., for nausea and vomiting.

Second Case—Mr. G. was attacked, and treated same as Mrs. B. Had Hæmaturia; was delirious; recovered.

Third Case—Julia J. was attacked with fever after a violent chill. Vomited dark brownish green matter. Symptoms and treatment same as in the first case. Blistered; serum nearly black; some hiccupping; constant nausea; tongue dry; *no hæmaturia.* Attacked September 29; died October 13. Had small purplish spots on face and neck three days before death; larger spots or splotches appeared on trunk and limbs day before death.

Fourth Case—B. two years old; had been having chills. March 21st had a chill more violent than any before; found him very much prostrated. After reaction pulse was small, quick and wiry. Treated him same as the others. Skin became yellow; was delirious. Died next day at the time for recurrence of chills. No hæmaturia.

Remarks.—In all these cases the patients were living in a malarial region; had been suffering with attacks of intermittent fever; the attack in which I was called was more violent than any preceding. In every case there was great prostration and constant irritability of the stomach; pulse in all small, wiry and quick; hæmaturia in two of the cases, not in the other two; two had delirium, one of whom recovered; the others were rational to within a very short time of their death. There was very little variation in the treatment, as the conditions preceding the attack and the attending symptoms were nearly identical in the four cases. It will be observed that the patients had all been suffering with chills prior to their last attack; that the yellowness of surface did not usually appear before the second or third day; at least not enough to attract attention. The bloody urine also appeared after some days' continuance of fever.

I have heard this disease called yellow fever, hemorrhagic fever, etc., but believe it to be a high grade of bilious fever, and have spoken of it as congestive fever, and again as malignant bilious fever. The cases occurred amongst a class of people in which it was impossible to have a post mortem examination, consequently we have no positive knowledge of the organs most affected, nor how they were affected. I believe there had been a congestive chill, in which the liver bore the burden of the congestion; that the congestion resulted in only impaired function, or may have amounted to disorganization of a smaller or greater portion of the organ, in which condition the blood was not prôperly elaborated to perform its offices in the economy. The kidneys, not having healthy blood presented for their peculiar action, receive the thin diseased blood, which, not being properly acted on by them, fails to pursue its normal course; the circulation becomes impeded, and congestion and exudation, or extravasation result; hence the bloody urine. Besides, it is well known that the kidneys, by a vicarious action, remove the most poisonous of the retained biliary matters, and this increased duty imposed on the kidneys will also tend to congestion and favor hæmaturia; and the depraved or imperfect condition of the blood will also account for the purpuric spots.

Dr. J. C. B. Williams (p. 253 Carpenter's Physiology,) mentions a case of albuminuria in which "the coloring matter of the blood was found dissolved in the liquor sanguinis, scarcely any perfect corpuscles being left. He has observed a similar total destruction of blood discs in a case of malignant scarlatina with purpura, and has met with the same in acute purpura *connected with jaundice*, and in cases of *functional derangement of the liver.*"

I have been asked if I did not think, if my views were correct, that tincture of iron would be a good remedy. I replied, yes, providing the liver was not so seriously damaged as to preclude the possibility of recovery, and the disease was recognized in time. But in my cases the hæmaturia appeared so late that I had to manage them without it. If I met with hæmaturia, or other hemorrhage, early in the attack I would give tincture of iron. Since this conversation with Drs. W. L. Davis and W. P. Jennings, of Albany, Georgia, Dr. Davis informed me that he had treated one patient in three distinct attacks with tincture of iron; and Dr. Jennings told me in October that he had treated a case (Mrs. B.) with tincture of iron; both recovered. They of course used the tincture of iron after a mercurial cathartic, and while giving quinine freely. I believe the hæmaturia to be accidental, two only of the four cases described having had it, and not of sufficient importance to justify the introduction of a new name for the affection.

In writing this paper I have refrained as much as possible from theorizing or entering upon the consideration of the peculiar action of the liver and kidneys on the blood, and have made but one quotation to show that a similar vitiated condition of the blood had been noticed in connection with disordered function of the liver.

